# First‐in‐Human Regulatory T Cell‐Derived Exosomes Plus Ranibizumab for Refractory nAMD

**DOI:** 10.1002/jev2.70396

**Published:** 2026-11-05

**Authors:** Ying Tian, Zhuyun Qian, Shuzhen Fan, Jianmin Wang, Wei Wei, Yong Tao

**Affiliations:** ^1^ Department of Ophthalmology Beijing Chaoyang Hospital Capital Medical University Beijing P.R. China; ^2^ Key Laboratory Jointly Built by the National Institute for Viral Disease Control and Prevention of China Centre for Disease Control and Prevention Beijing GIANTMED Medical Diagnostics Laboratory Beijing P.R. China; ^3^ Department of Ophthalmology Hebei General Hospital Hebei P.R. China; ^4^ State Key Laboratory of Biopharmaceutical Preparation and Delivery Institute of Process Engineering Chinese Academy of Sciences Beijing P.R. China; ^5^ School of Chemical Engineering University of Chinese Academy of Sciences Beijing P.R. China; ^6^ National Engineering Research Centre for Ophthalmology Beijing P.R. China; ^7^ Engineering Research Centre of Ophthalmic Equipment and Materials Ministry of Education Beijing P.R. China; ^8^ Chongqing Medical University Eye Institute & Chongqing Institute of Intelligent Medicine Chongqing China

## Abstract

Persistent fluid despite intravitreal anti–vascular endothelial growth factor (VEGF) treatment remains common in neovascular age‐related macular degeneration (nAMD). Regulatory T cell–derived exosomes (TregExo) have preclinical immunomodulatory activity. To evaluate the safety and maximum tolerated dose (MTD) of intravitreal autologous TregExo plus ranibizumab in refractory nAMD. Design, Setting, and Participants: Single‐centre, prospective, open‐label phase 1a trial using a 3+3 dose‐escalation design at Hebei General Hospital. Four participants with persistent intraretinal or subretinal fluid despite at least 3 consecutive monthly intravitreal anti‐VEGF injections were treated. One intravitreal injection of autologous TregExo (planned doses, 0.05, 0.5, or 1.5 mg) coformulated with ranibizumab, 0.5 mg. The primary outcome was safety and MTD determination. Dose‐limiting toxicity (DLT) was prespecified as severe sterile intraocular inflammation, sustained intraocular pressure greater than 30 mm Hg, loss of more than 15 ETDRS points, or another serious ocular adverse event within 30 days. Exploratory visual and anatomic outcomes were assessed through day 90. Four participants were treated: 3 at 0.05 mg and 1 at 0.5 mg. No DLT or intraocular inflammation occurred in the 0.05‐mg cohort, and intraocular pressure remained stable. Two low‐dose participants improved from ETDRS scores of 3 and 9 at baseline to 35 at day 90; Patient 1 had a macular thickness reduction from 318.2 to 253.9 µm. The 0.5‐mg participant developed sterile intraocular inflammation with vision loss and a large intraretinal cyst, which resolved after local corticosteroid therapy. Dose escalation was stopped, and 0.05 mg was the protocol‐defined MTD. Intravitreal autologous TregExo plus ranibizumab had an identifiable dose ceiling: 0.05 mg was tolerated, whereas 0.5 mg was associated with DLT. Further controlled evaluation of lower‐dose TregExo‐based adjunctive therapy in refractory nAMD is warranted.

**Trial Registration**: Chinese Clinical Trial Registry Identifier: ChiCTR2500095798.

## 1 Introduction

Neovascular age‐related macular degeneration (nAMD) is a leading cause of central vision loss, and intravitreal anti‐vascular endothelial growth factor (VEGF) therapy remains the standard treatment. Despite this advance, persistent or recurrent intraretinal or subretinal fluid remains common after repeated injections, leaving a clinically important subgroup with refractory disease (Yang et al. 2016, Mettu et al. 2021, Broadhead et al. 2014). The need for repeated treatment also increases visit burden for patients and clinics (Mettu et al. 2021). Switching anti‐VEGF agents can improve anatomy in some eyes, but long‐term gains are inconsistent because these agents remain cantered on VEGF‐pathway inhibition (Ashraf et al. 2018). Dose escalation with high‐dose aflibercept can improve retinal drying and extend treatment intervals in some patients, yet refractory exudation still persists in a subset (Wykoff et al. 2023).

The current benchmark for moving beyond single‐pathway VEGF inhibition is faricimab, which targets both angiopoietin‐2 and VEGF‐A (Heier et al. 2022). In phase 3 trials and subsequent real‐world reports, faricimab allowed longer treatment intervals and helped some refractory eyes, but persistent fluid still occurs in a clinically meaningful subset (Heier et al. 2022, Yufeng et al. 2025). Other adjunctive strategies, including photodynamic therapy, radiotherapy, and anti‐platelet‐derived growth factor combinations, have not shown consistent benefit beyond anti‐VEGF monotherapy (Gao et al. 2018, Evans et al. 2020, Jackson et al. 2023, Arepalli and Kaiser 2021). This pattern suggests that refractory nAMD is mechanistically heterogeneous rather than simply undertreated.

One plausible contributor is inflammation. Experimental and clinical data implicate cytokine‐driven and macrophage‐related signaling in choroidal neovascularization and incomplete anti‐VEGF response (Zhao and Lai 2024). In preclinical models, regulatory T cell‐derived exosomes (TregExo) reduced both inflammatory and angiogenic activity in choroidal neovascularization (Tian et al. 2021). We therefore conducted a first‐in‐human phase 1a dose‐escalation trial to evaluate the safety of intravitreal autologous TregExo combined with the anti‐VEGF agent ranibizumab in refractory nAMD. The treatment landscape relevant to this study is summarized in Figure 1.

**FIGURE 1:**
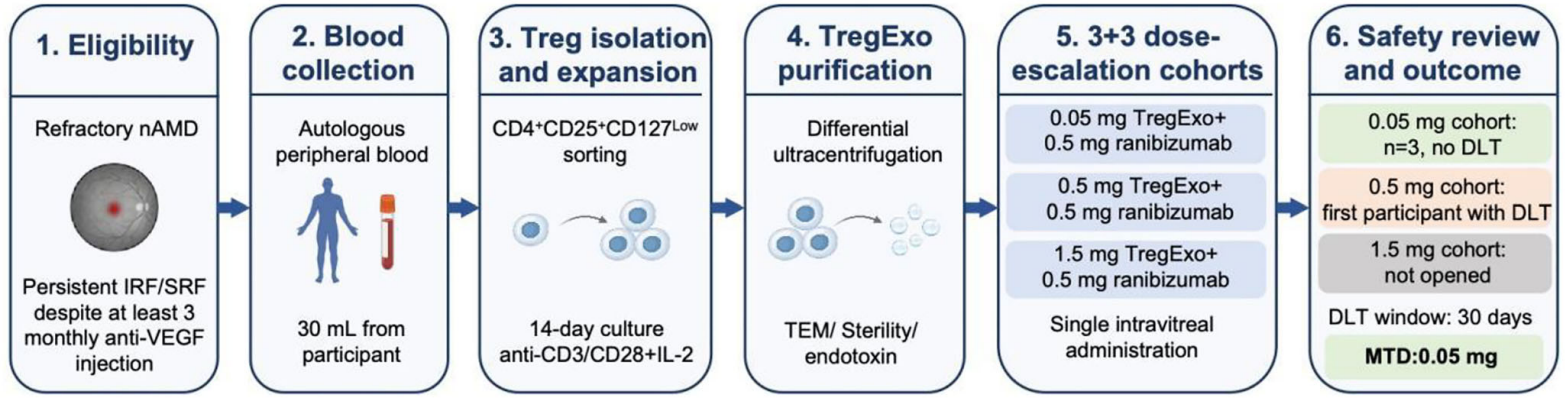
Trial schema for intravitreal autologous TregExo plus ranibizumab in refractory nAMD. The figure shows eligibility, autologous blood collection, Treg isolation and expansion, TregExo purification, planned 3+3 dose‐escalation cohorts, and safety review, with 0.05 mg identified as the maximum tolerated dose.

## 2 Methods

This single‐centre, prospective, open‐label, investigator‐initiated phase 1a trial (ChiCTR2500095798) used a standard 3+3 dose‐escalation design. The study was approved by the Medical Ethics Committee of Hebei General Hospital (No. 2024‐409‐1), and all participants provided written informed consent. Participants were recruited between December 2024 and December 2025.

Eligible participants had refractory nAMD with persistent intraretinal fluid or subretinal fluid on spectral‐domain OCT despite at least 3 consecutive monthly intravitreal anti‐VEGF injections. Prior anti‐VEGF treatment histories were documented for each participant (Table S1). Exclusion criteria were intravitreal anti‐VEGF therapy, verteporfin photodynamic therapy, or macular laser photocoagulation within 1 month before screening; dexamethasone implant within 6 months or periocular long‐acting corticosteroids within 3 months; and major ocular surgery or any intraocular surgery within 3 months before screening.

The investigational product was manufactured from 30 mL of autologous peripheral blood. CD4^+^CD25^+^CD127^low^ regulatory T cells were isolated by magnetic bead sorting, expanded for 14 days in a good manufacturing practice‐compliant facility with anti‐CD3/CD28 antibodies and recombinant human interleukin‐2. Immediately before EV production, the viability of expanded Tregs was determined by trypan blue exclusion, and their phenotype was assessed by flow cytometry for CD4, CD25, and CD127 (Figure S1 and Figure 2A). These cells were then used to generate exosomes, which were harvested by differential ultracentrifugation in keeping with MISEV2023 recommendations (Welsh et al. 2024). Vesicle morphology was confirmed by transmission electron microscopy, and particle size distribution and zeta potential were characterized by nanoparticle tracking analysis (Figure 2B,C). Western blotting confirmed the presence of CD63, ALIX, and TSG101 and the absence of calnexin in representative TregExo preparation (Figure S2). Before administration, each TregExo preparation was required to meet the predefined release criteria of a negative sterility test and an endotoxin level of <0.2 EU/dose (Table S2). Manufacturing parameters and quality‐control results were consistent across the clinical batches (Table S3). Unmixed TregExo was transported on dry ice, stored at −80°C, and used within 7 days during which its particle size and zeta potential remained stable (Figure S3), and ranibizumab was added immediately before injection with compatibility supported by unchanged protein profiles and particle‐size distributions (Figure S4).

**FIGURE 2:**
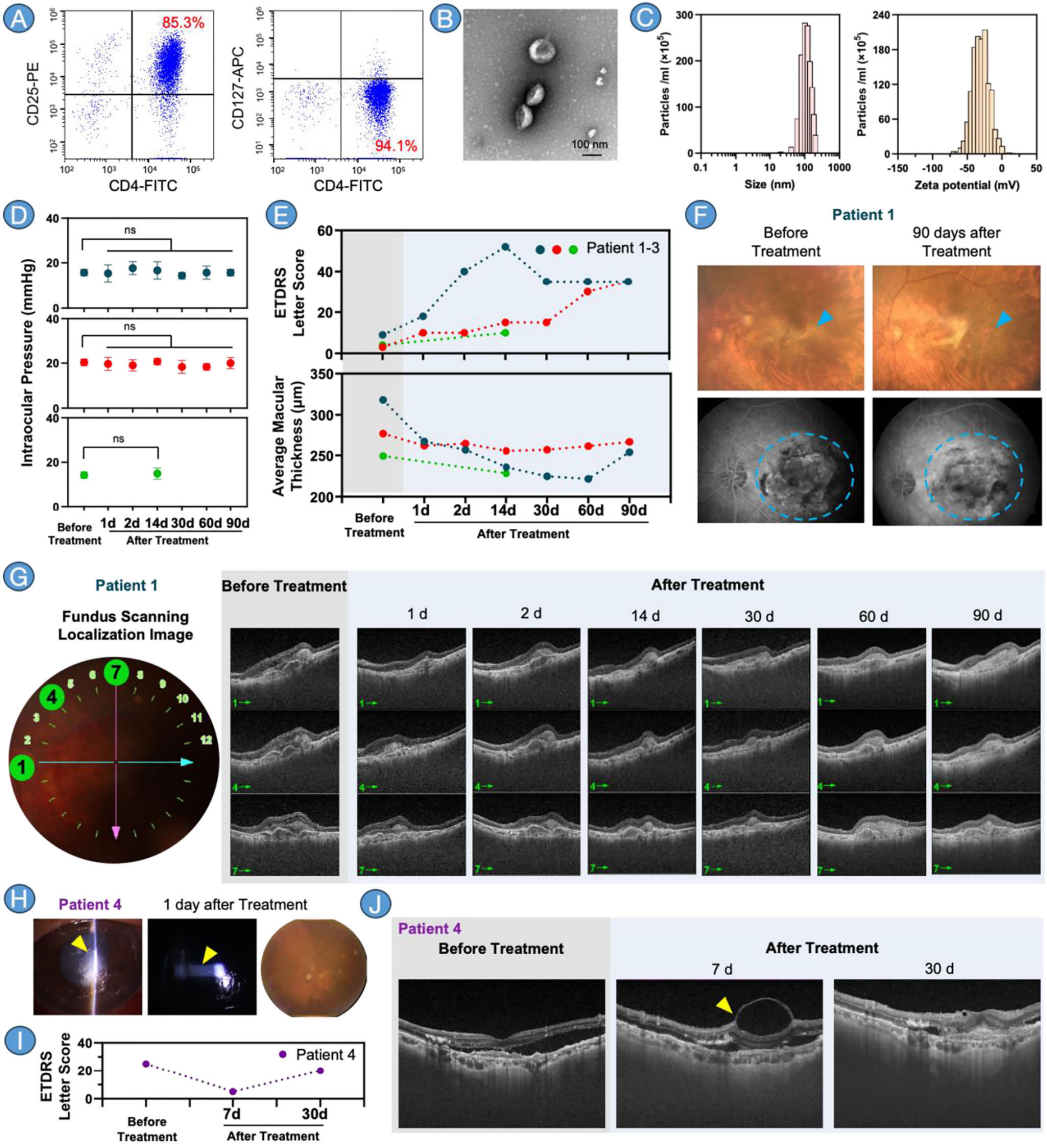
Phase 1a dose‐escalation trial of autologous TregExo plus ranibizumab for refractory nAMD. (A). Flow cytometry of expanded regulatory T cells showing CD4^+^CD25^+^ and CD4^+^CD127^low^ populations. (B). Transmission electron microscopy image showing the characteristic cup‐shaped morphology of the TregExo. (C). Nanoparticle tracking analysis showing particle size distribution within the TregExo range and zeta potential. (D). IOP values in the 0.05‐mg cohort (*n* = 3) during follow‐up. (E). Longitudinal ETDRS visual acuity and OCT macular thickness in the 0.05‐mg cohort. (F). Representative colour fundus photography and fluorescein angiography from Patient 1 at baseline and day 90 showing reduced exudation and leakage. (G). Serial OCT images from Patient 1 showing reduced exudative lesion burden over time. (H). Representative anterior segment and fundus images from the first participant treated at 0.5 mg showing acute sterile intraocular inflammation on day 1. (I). Longitudinal ETDRS visual acuity in the 0.5‐mg participant. (J). Serial OCT images from the 0.5‐mg participant showing a large intraretinal cyst at day 7 with subsequent resolution after treatment.

TregExo dose was defined by total protein content. Participants received a single intravitreal injection of TregExo at 0.05 mg, 0.5 mg, or 1.5 mg, each coformulated with ranibizumab, 0.5 mg, in 100 µL of sterile buffer. Anterior chamber paracentesis was performed in all participants before injection to reduce the risk of acute intraocular pressure (IOP) elevation. The primary outcome was safety and MTD determination. DLT was prespecified as severe sterile intraocular inflammation, sustained IOP greater than 30 mmHg, a decrease of more than 15 points in ETDRS score from baseline, or another serious treatment‐related ocular adverse event occurring within 30 days after injection. Exploratory outcomes included ETDRS visual acuity, macular thickness on OCT, and fluorescein angiographic leakage through postoperative day 90.

Graph generation and statistical analysis were performed using GraphPad Prism 9.5. IOP is presented as mean ± SD and analysed using one‐way ANOVA or Student's t‐test, with *P* < 0.05 considered statistically significant. Due to the small sample size, other clinical outcomes are presented descriptively without statistical testing.

## 3 Results

Four participants were treated and no paracentesis‐related complications occurred. Three participants received 0.05 mg of TregExo plus ranibizumab and completed 90 days of follow‐up. No DLTs or signs of intraocular inflammation were observed in this cohort, and IOP remained stable (Figure 2D). Exploratory improvements were seen in both visual and anatomic measures. Two participants improved from baseline ETDRS scores of 3 and 9 to 35 at day 90. Patient 1 had a reduction in macular thickness from 318.2 µm at baseline to 253.9 µm at day 90, and serial OCT and fluorescein angiography suggested decreased exudative activity (Figure 2E–G). Serial OCT imaging showed that macular thickness decreased from 276.5 µm at baseline to 266.5 µm at day 90 in Patient 2 and from 249.4 µm at baseline to 228.3 µm at day 14 in Patient 3 (Figure 2E and Figure S5A,SB).

After the low‐dose cohort completed protocol review, dose escalation proceeded to 0.5 mg. The first participant treated at this dose developed acute sterile intraocular inflammation on day 1, with corneal epithelial detachment, anterior chamber flare, and vitreous opacity (Figure 2H). By day 7, this participant had marked vision loss and a large intraretinal cyst in the macular region. Local corticosteroid therapy was followed by clinical resolution, supporting classification of the event as sterile inflammation (Figure 2I, J and Figure S5C). Dose escalation was stopped, the 1.5‐mg cohort was not opened, and 0.05 mg was designated the protocol‐defined MTD.

## 4 Discussion

This first‐in‐human phase 1a trial identifies a narrow ocular dose window for intravitreal autologous TregExo plus ranibizumab in refractory nAMD. The central finding is the coexistence of acceptable short‐term tolerability at 0.05 mg during the prespecified 30‐day DLT assessment window and DLT at 0.5 mg. That distinction is important because it defines the safety boundary for future studies of this platform.

This study helps position TregExo plus ranibizumab within the current treatment landscape for refractory nAMD. In eyes with persistent exudation despite prior anti‐VEGF therapy, available strategies include switching agents, dose escalation, dual‐pathway blockade, and other adjunctive combinations. Against that background, TregExo plus ranibizumab is best understood as a first‐in‐human immunomodulatory adjunctive strategy rather than as a direct substitute for an established therapy, as shown in Table 1.

**TABLE 1:** Clinical Context of TregExo Plus Ranibizumab in Refractory nAMD.

Strategy	Evidence	Potential Benefit	Key Limitation	Relevance
Switching anti‐VEGF agents	Reviews of refractory nAMD and switching studies (Broadhead et al. 2014, Ashraf et al. 2018)	Anatomic improvement in some patients	Still targets the VEGF axis	Supports testing a non‐VEGF adjunct
Dose escalation	High‐dose aflibercept trial (Wykoff et al. 2023)	Better drying and longer intervals in some patients	Residual exudation in a subset	Suggests refractoriness is not explained by dose alone
Dual‐pathway blockade with faricimab	TENAYA/LUCERNE and real‐world reports (Heier et al. 2022, Yufeng et al. 2025)	Longer intervals and benefit in some refractory eyes	Persistent fluid despite Ang‐2/VEGF‐A blockade	Current comparator beyond single‐pathway anti‐VEGF therapy
Prior adjunctive combination approaches	PDT meta‐analysis, radiotherapy review, MERLOT, and anti‐PDGF review (Gao et al. 2018, Evans et al. 2020, Jackson et al. 2023, Arepalli and Kaiser 2021)	Showed the feasibility of combination therapy	No consistent visual advantage over anti‐VEGF monotherapy	Suggests success may require an independent target
TregExo plus ranibizumab	Preclinical TregExo work and current trial (Tian et al. 2021)	Combines anti‐VEGF therapy with immunomodulation	Safety boundary and clinical benefit remain to be established	Positions this study as a first‐in‐human adjunctive approach in refractory nAMD

This study has several limitations. It was conducted at a single centre, included only 4 treated participants, and had no control group. Because all participants also received ranibizumab, the exploratory visual and anatomic changes cannot be attributed to TregExo alone. In addition, TregExo dosing was based on total protein, which may not fully reflect the biologically active EV dose; future dose‐optimization studies should incorporate particle number, particle‐to‐protein ratio, and potency assays as complementary metrics. Larger controlled studies will be needed to determine whether a lower‐dose TregExo regimen can improve outcomes beyond standard anti‐VEGF therapy in refractory nAMD.

## Author Contributions

**Ying Tian**: writing – original draft, data curation, investigation, formal analysis. **Zhuyun Qian**: software, investigation. **Shuzhen Fan**: data curation. **Jianmin Wang**: methodology, supervision, funding acquisition. **Wei Wei**: supervision, methodology, funding acquisition. **Yong Tao**: conceptualization, project administration, writing – review and editing, funding acquisition, supervision.

## Conflicts of Interest

The authors declare no conflicts of interest.

## Supporting information




**Supporting Information**: jev270396‐sup‐0001‐SuppMat.docx

## Data Availability

The data that support the findings of this study are available from the corresponding author upon reasonable request.
