# Supplementary material for: First‐in‐Human Regulatory T Cell‐Derived Exosomes Plus Ranibizumab for Refractory nAMD
Source: J Extracell Vesicles. 2026 Nov 5;15(11):e70396. doi: 10.1002/jev2.70396 (PMC13739763; doi:10.1002/jev2.70396)
Supplement: Supplementary file 1 [file JEV2-15-e70396-s001.docx]

****Supplementary Materials****

****First-in-Human Regulatory T Cell-Derived Exosomes Plus Ranibizumab for Refractory nAMD****

****Contents****

**Supplementary Figure 1. Trypan blue–based viability assessment of expanded Tregs before EV production.**

**Supplementary Figure 2. Western blot characterization of TregExo.**

Supplementary Figure 3. Stability of TregExo during storage at −80°C.

Supplementary Figure 4. Physicochemical compatibility of TregExo and ranibizumab.

Supplementary Figure 5. Serial optical coherence tomography images of Patients 2–4 before and after treatment.

**Supplementary Table 1. Prior anti-VEGF treatment history of each participant**

**Supplementary Table 2. Release criteria and additional quality characterization of the TregExo preparations**

**Supplementary Table 3. Manufacturing reproducibility, batch-specific quality control**

****Supplementary Figure 1. Trypan blue–based viability assessment of expanded Tregs before EV production.****

(A) **Representative images of trypan blue-stained expanded Tregs before EV production, showing the raw cell image (left) and software-assisted cell classification (right). Viable and nonviable cells are indicated by green and red outlines, respectively. Scale bar, 100 μm.**
(B) **Quantification of expanded Tregs viability immediately before EV production. Data are presented as mean ± s.d. (n = 3)**

**The mean viability of expanded Tregs immediately before EV production was 95.8% ± 1.4%.**

****Supplementary Figure 2. Western blot characterization of TregExo.****

(A) **Western blot analysis of CD63, ALIX, TSG101, calnexin, and β-actin in the control and TregExo samples. R1 and R2 represent technical replicates of the same TregExo sample.**
(B) **Original uncropped blots corresponding to the cropped images shown in (A).**

**CD63, ALIX, and TSG101 were detected in TregExo, whereas calnexin was not detected.**

**Supplementary Figure 3. Stability of TregExo during storage at −80°C.**

Mean particle size (red line, left y-axis) and zeta potential (green line, right y-axis) were measured at days 0, 3, 7, 14, and 21. Data are presented as mean ± SD (n = 3).

Mean particle size and zeta potential remained comparable to baseline throughout the 21-day storage period.

**Supplementary Figure 4. Physicochemical compatibility of TregExo and ranibizumab.**

(A) SDS–PAGE analysis of ranibizumab alone (lane 1), TregExo alone (lane 2), the TregExo–ranibizumab mixture immediately after preparation (lane 3), and the mixture after incubation for 2 h at 4 ℃ (lane 4).
(B) Particle-size distributions of the TregExo–ranibizumab mixture immediately after preparation (left) and after incubation for 2 h at 4 ℃ (right).

No readily detectable changes in the protein profile or particle-size distribution were observed after incubation for 2 h at 4 ℃.

**Supplementary Figure 5. Serial optical coherence tomography images of Patients 2–4 before and after treatment.**

(A) Patient 2 at baseline and days 1, 2, 14, 30, 60, and 90 after treatment.
(B) Patient 3 at baseline and day 14 after treatment.
(C) Patient 4 at baseline and days 7 and 30 after treatment.

Serial OCT imaging showed reduced macular thickness in Patients 2 and 3, whereas the large intraretinal cyst observed in Patient 4 on day 7 showed marked resolution by day 30.

****Supplementary Table 1. Prior anti-VEGF treatment history of each participant****

| Patient ID | Study eye | Dose cohort | Prior anti-VEGF agent | No. of previous injections | Interval from last anti-VEGF injection to study treatment |
| --- | --- | --- | --- | --- | --- |
| 1 | Left | 0.05 mg TregExo  +0.5 mg ranibizumab | Ranibizumab | 3 | 2 years |
| 2 | Left | 0.05 mg TregExo  +0.5 mg ranibizumab | Aflibercept | 10 | 12 weeks |
| 3 | Left | 0.05 mg TregExo  +0.5 mg ranibizumab | Ranibizumab | 4 | 8 weeks |
| 4 | Left | 0.5 mg TregExo  +0.5 mg ranibizumab | Aflibercept | 3 | 1 year |

****Supplementary Table 2. Release criteria and additional quality characterization of the TregExo preparations****

| Classification | Quality attribute | Analytical method | Predefined acceptance criterion |
| --- | --- | --- | --- |
| Formal release criterion | Sterility | Sterility test | Negative |
|  | Endotoxin | Endotoxin assay | <0.2 EU/dose |
| Product characterization | Vesicle morphology | TEM | Cup-shaped morphology |
|  | Particle size distribution | NTA | 30 to 200 nm |
|  | Zeta potential | NTA | −10 to −50 mV |
| Dose-defining attribute | Total protein content | Microvolume UV–visible spectrophotometry | Protocol-specified target protein dose |

TEM: Transmission Electron Microscopy, NTA: Nanoparticle Tracking Analysis

****Supplementary Table 3. Manufacturing reproducibility, batch-specific quality control****

| Batch  NO. | Patient ID | Blood volume | Treg expansion period | Mean particle size | Mean  Zeta potential | Sterility | Endotoxin | Utilized  TregExo dose |
| --- | --- | --- | --- | --- | --- | --- | --- | --- |
| Batch 1 | 1 | 30 mL | 14 days | 120.3 nm | -32.8 mV | Negative | <0.2 EU/dose | 0.05 mg |
| Batch 2 | 2 | 30 mL | 14 days | 111.0 nm | -37.6 mV | Negative | <0.2 EU/dose | 0.05 mg |
| Batch 3 | 3 | 30 mL | 14 days | 122.4 nm | -37.2 mV | Negative | <0.2 EU/dose | 0.05 mg |
| Batch 4 | 4 | 30 mL | 14 days | 118.9 nm | -38.9 mV | Negative | <0.2 EU/dose | 0.5 mg |
